# Evaluating Community Partnerships Addressing Community Resilience in Los Angeles, California

**DOI:** 10.3390/ijerph15040610

**Published:** 2018-03-27

**Authors:** Malcolm V. Williams, Anita Chandra, Asya Spears, Danielle Varda, Kenneth B. Wells, Alonzo L. Plough, David P. Eisenman

**Affiliations:** 1 RAND Corporation, Santa Monica, CA 90401, USA; aspears@rand.org (A.S.); kwells@mednet.ucla.edu (K.B.W.); deisenman@mednet.ucla.edu (D.P.E.); 2 RAND Corporation, Arlington, VA 22202, USA; chandra@rand.org; 3 School of Public Affairs, University of Colorado Denver, P.O. Box 173364, Denver, CO 80217, USA; danielle.varda@ucdenver.edu; 4 West Los Angeles VA Healthcare Center, Los Angeles, CA 90073, USA; 5 Department of Psychiatry and Biobehavioral Sciences, David Geffen School of Medicine at UCLA, Los Angeles, CA 90095, USA; 6 Center for Public Health and Disasters, UCLA Fielding School of Public Health, Los Angeles, CA 90095, USA; 7 Robert Wood Johnson Foundation, Princeton, NJ 08540, USA; aplough@rwjf.org; 8 Division of General Internal Medicine, David Geffen School of Medicine at UCLA, Los Angeles, CA 90095, USA

**Keywords:** community resilience, disaster preparedness, community coalitions, social network analysis, public health nursing, disaster risk reduction, public health practice

## Abstract

Community resilience has grown in importance in national disaster response and recovery efforts. However, measurement of community resilience, particularly the content and quality of relationships aimed at improving resilience, is lacking. To address this gap, we used a social network survey to measure the number, type, and quality of relationships among organizations participating in 16 coalitions brought together to address community resilience in the Los Angeles Community Disaster Resilience project. These coalitions were randomized to one of two approaches (community resilience or preparedness). Resilience coalitions received training and support to develop these partnerships and implement new activities. Both coalition types received expert facilitation by a public health nurse or community educator. We also measured the activities each coalition engaged in and the extent to which partners participated in these activities at two time points. We found that the community resilience coalitions were initially larger and had lower trust among members than the preparedness communities. Over time, these trust differences dissipated. While both coalitions grew, the resilience community coalitions maintained their size difference throughout the project. We also found differences in the types of activities implemented by the resilience communities; these differences were directly related to the trainings provided. This information is useful to organizations seeking guidance on expanding the network of community-based organizations that participate in community resilience activities.

## 1. Introduction

Community resilience, or the sustained ability of a community to withstand and recover from adversity (e.g., economic stress, man-made or natural disaster), continues to be a key global policy issue [1,2,3,4] that has grown in interest around the world [4,5,6], particularly in the context of Hurricanes Harvey, Irma, and Maria in the U.S.; the Japanese earthquake and Tsunami of 2011; the Haiti earthquake of 2010; the Canterbury Earthquakes of 2010–2011 in New Zealand; and the Indian earthquake and Tsunami of 2005 internationally. The application of community resilience to disaster planning and response must account for variability in the underlying contexts and resources of communities and the resulting differences in their capacity to embrace resilience constructs. However, as Villagra and Quintanait [7] described, it has nevertheless emerged as one of the most important frameworks for community level approaches to disaster planning [7,8].

The concept of resilience has its roots in the fields of psychology and ecology where it has been used to describe individual or system level adaptability in the face of a threat. Resilience was originally applied to emergency preparedness contexts to describe how physical infrastructures might withstand and recover from a disaster [9]. Over several decades, the definition of community resilience has been expanded to include a range of concepts related to the community’s ability to deal with disasters including community planning, mitigation, recovery, the development of networks, and the ability of a community to learn from prior events [9]. Taken together, community resilience focuses on developing new capabilities among emergency planners, urban planners, community leaders, and public health providers to leverage community assets and integrate community resources into preparedness planning and response to address a range of shocks and stresses [10]. In this way, community resilience represents a new orientation for public health preparedness, as it bridges the emergency management framework, which initially almost exclusively shaped public health emergency preparedness (PHEP) efforts within Local Health Departments (LHDs) and the more traditional, but often separate, public health promotion activities characterized by community engagement methods. Bridging of PHEP and public health promotion produces opportunities for synergy and dual benefit in how public health activities are implemented. Building community resilience requires a much broader integration of preparedness across LHD activities, thus potentially integrating all community engagement activities across an entire department. This may include developing collaborations between emergency preparedness and other health department programs such as maternal and child health, chronic disease, as well as partnerships between health departments and other existing community-based organizations working on health promotion or other community initiatives within or outside of health [11,12].

Relying on community-based relationships to build community resilience is important in part because leveraging outside resources can enhance or extend the reach of public health activities while not taxing an already stressed and limited formal workforce [13,14]. Such partnerships between LHDs and community-based organizations (e.g., churches, schools, nursing homes) increase the capacity of LHDs to provide public health services and, in some cases, reach marginalized communities [15]. An emphasis on partnering with community-based organizations (CBOs) is important because these organizations are a critical component of the public health systems [16], have the capabilities needed to provide public health interventions, and are trusted resources in their communities. Indeed, building community resilience requires connecting community members, community-based organizations, businesses, and government agencies across sectors. For instance, the Sendai Framework calls for “all-of-society engagement and partnership” with a focus on “the improvement of organized voluntary work of citizens” [17]. The United Nations International Strategy for Disaster Reduction (UNISDR) [18] and the Rockefeller 100 Resilient Cities Initiative also emphasize the importance of multi-sector partnerships.

Despite these advantages, there are many challenges to developing such partnerships including lack of funding, limited staff availability, and the difficulty of strengthening and expanding partnerships beyond a specific health issue or intervention [19]. Early research on the Los Angeles County Health Department’s resilience efforts found that staff were challenged by a lack of training in how to effectively engage community partners. In addition, despite the growing emphasis on community partnerships, little is known about their impact on community resilience goals. For example, several case studies have identified barriers to such partnerships including difficulties with integrating public and private systems, uncertainty about the roles of partners, expectations about the outcomes of the partnership, accountability, and in some cases low trust among partners [20,21,22]. Early research on the Los Angeles County Health Department’s resilience efforts found that staff were challenged by a lack of training in how to effectively engage community partners [23]. Thus, the process of building community resilience raises numerous “how to” questions, such as how to strengthen a community’s ability to be resilient to health security emergencies and how to measure progress towards achieving that end goal. As LHDs and their partners work to enhance community resilience, these organizations may benefit from examples and guidance on how to translate the conceptual view of community resilience into an operational one, with attention to the optimal ways to build stronger collaborations to develop resilience capabilities.

Measurement of resilience is particularly problematic. There are both community resilience frameworks [4,24,25] and conceptual models [26] that can guide resilience evaluation approaches generally. However, while partnerships are clearly an important element of building resilience [4,14,27,28] there are not clear metrics for measuring these relationships.

Further, partnerships are highly variable with limited data on what makes them effective. Per recent data from the U.S. National Association of City and County Health Officials (NACCHO), the clear majority of LHDs partner with community and faith-based organizations (CBOs). However, their data do not assess the strength and effectiveness of these links [29]. This gap reflects a much broader measurement challenge with respect to understanding and tracking effective partnerships. Typical partnership evaluation tools used by LHDs often include only a broad-based qualitative assessment of partnerships at the aggregate level [29]. General open-ended questions such as these tend not to not allow for respondents to delineate among various partnership members and their individual contributions and impacts, along domains of partnership relevant to a given issue such as preparedness. In other words, these assessments do not provide information about how the relationships among each set of partners affect the larger collaborative goal, nor do they allow analysis of how activities such as sharing resources or information within the collaboration benefit (or hinder) the efforts to achieve the partnership goal. A better understanding is needed of how these challenges can be overcome and what makes partnerships succeed. In short, measurement of partnerships and their outcomes is critically needed.

In summary, community resilience as an organizing frame for engagement activities has great potential to integrate health promotion activities in communities and provide improved approaches for partnership development. However, little is currently known about what makes LHD-CBO partnerships work in the context of building resilience for disaster preparedness. For example, it is not clear if it is better to have more or fewer partners working on the same activities, or if diversity in terms of sectors improves coalition functioning. Moreover, we know very little about the quality of these collaborative relationships and the characteristics that support a collaborative’s ability to effect measurable change in a relevant health or community resilience improvement outcome. For example, it is not clear whether relationship attributes such as trust affect how coalition members frame or accomplish their resilience goals. Further, as emergency planners work to implement community resilience strategies, they are confronted with the problem of how to demonstrate improvement in resilience capacities and capabilities when such measures in relation to coalitions are relatively nascent.

Given that a resilient community is dependent on strong networks that can help in disaster preparedness and response [7,27], it is critical to understand how public health collaboratives can improve their partnerships specifically to meet the unique needs of resilience building. Our study endeavors to address this gap by measuring the organizational networks of 16 community coalitions developed to build community resilience via a large demonstration project conducted in Los Angeles County, California [10,30,31,32]. By doing so, we are seeking to refine specific partnership measures used to assess resilience capacities and activities within the coalition and provide objective information about their growth and development over time.

The specific research questions we sought to address were the following:

- How do coalitions of community partners, engaged in building community resilience for disasters as an approach to preparedness, develop and expand over time?
- What is the quality of these community partnerships, and how do they change over the course of time?
- What strategies do these coalitions use to achieve specific disaster resilience outcomes?
- How do coalitions differ in their partnership structure and strategies over time, as a function of intervention support from LHDs (for either implementing more traditional, expert-driven preparedness activities, or for an intentional, community resilience approach using community engagement as a lever for partnership development)?

## 2. Materials and Methods

As previously reported, between 2010 and 2014 the Los Angeles County Department of Public Health (LACDPH) developed and piloted an approach called the Los Angeles County Community Disaster Resilience Project (LACCDR) [10,30,31,32], which was rooted in developing community resilience capability in four levers of a resilience framework developed by Chandra and colleagues [4]—community self-sufficiency; integrated partnerships among government and nongovernmental organizations; engagement of at-risk populations in resilience planning; and education of all populations about preparedness, response, and recovery.

A focal point of the LACDPH resilience building efforts was to develop partnerships with CBOs to integrate individual communities into the disaster response and recovery framework of the LACDPH. Stakeholders were engaged early in the project through community forums, working groups, and community surveys to design the intervention [31]. The LACDPH randomly assigned 16 communities (two per Los Angeles County Service Planning Area (SPA)) to one of two conditions: community resilience vs. enhanced preparedness. The LACCDR then sought to identify existing coalitions with which to partner in each community to develop community-based activities centered around a community resilience or enhanced standard preparedness framework. Communities assigned to the community resilience condition arm (resilience communities) were provided access to a public health nurse to increase awareness of community resilience issues, build relationships among community leaders and other stakeholders, and to enhance the resources of community organizations to contribute to building resilience. The nurse was trained in these community resilience topics and facilitated the use of a community resilience toolkit among coalition members which addressed topics such as leadership development, asset mapping and social preparedness, community engagement processes, psychological first aid, developing field workers, and vulnerability assessment [32]. In addition to the targeted support through facilitated workshops centered on a toolkit and encouragement to broaden their coalitions, the resilience coalitions received explicit training on community resilience definitions and activities which included broadening the membership of the coalition to include a variety of partners. Building out the diversity in sectors represented in each resilience community was based on the Centers for Disease Control and Prevention (CDC) guidance on the 11 essential community sectors with which partnerships can improve population health, resilience, and extend the reach of public health. Among these sectors are emergency management, health care, social services, cultural and faith-based groups and organizations, businesses, community leadership, housing and sheltering, media, mental/behavioral health, organizations serving the interests of at-risk populations such as older persons, and education and childcare [11]. In short, the resilience communities were asked to grow their coalitions and reach larger numbers of sectors and organizations in the community to represent the various interests of a more diverse set of stakeholders, while building a content focus on disaster preparedness as a collaborative.

The comparison condition arm (which we label “preparedness”) had access to a community health educator who helped facilitate a standardized, organized form of public health preparedness practice focused mainly on improving household level preparedness with supplies and emergency plans [32]. These community health educators trained the preparedness coalitions on traditional preparedness, special populations, and strategies for linking with community groups. They were not formally trained in community resilience concepts and did not have access to the community resilience toolkit though they were exposed to some general aspects of the community resilience concept at annual overall project convenings and potentially through their pre-existing community health educator expertise.

### 2.1. Study Setting

The 8 LA County SPAs represent a diverse mix of communities. Several geographies exist among these regions, including coastal plain, mountain, and high desert. While densely populated urban communities dominate much of the county, there are also very large, sparsely populated, rural communities in SPA 1, which encompasses the high desert known as the Antelope Valley.

The stakeholder engagement process identified communities that fit the following criteria. Each community selected had to have a shared identity as a community, and, collectively, the communities had to reflect the diversity of the county’s demography, hazards, and geography. Each community was ideally modest in size (under 50,000) though one had greater than 100,000 persons [32]. Plus, each community had to have a sufficient basic infrastructure for developing a collaborative, including having a mix of stable community-based organizations and government institutions such as schools, police/fire departments, local businesses, and neighborhood councils.

### 2.2. Partnership Metrics

The primary goal of this research is to look closely at the organizational relationships and coalition activities that occurred in the LACCDR, assess change over time, and compare differences between the two arms especially with respect to achieving resilience goals. To accomplish this, we used a social network analysis tool called PARTNER [33] to measure the growth and development of the coalitions and their interactions in the 16 communities over a 12-month period.

PARTNER determines the key players in a network, the frequency of their interactions, and the context of their exchanges [33]. PARTNER is an online survey and analysis tool that can be customized with additional multiple choice and open-ended questions. These data permit us to make nuanced observations about relationships among organizations in each of the 16 coalitions, with information on the quality of the exchange relationships within a network, beyond describing how the network is organized or how frequently members meet. Information on the quality of the interactions among members and the meaning the members attribute to those exchanges, offer additional insights into how well the group or coalition is working toward its goals.

We measured community coalitions at two points in time spread approximately one year apart (May 2013–June 2014). To assess how the coalitions grew over time and any changes in the quality of their partnerships, we evaluated several factors identified as key in network analysis literature, including the number of organizations in a coalition, the number of CDC identified essential sectors represented by these organizations, and several aspects of their relationships with one another. The relationship characteristics have been previously validated [33] and include:

- Trust among partners (measured as an index of three questions asking about the extent to which each of the other organizations in the coalition is reliable, supports the mission of the coalition, and is open to discussion). Responses options are (1) not at all, (2) a small amount, (3) a fair amount, (4) a great deal.
- Perceived value of partners to the mission, measured as an index of three questions asking about each organization’s perception of the other partners as valuable to achieving the overall mission of the coalition in terms of power/influence, commitment, and resources available. Responses options are (1) not at all, (2) a small amount, (3) a fair amount, (4) a great deal.
- Density or the number of connections reported between organizations as a function of all possible connections; lower density suggests that there are greater opportunities to increase connections among partners within a coalition.
- Activity coordination is measured along a four-level continuum (moving from lower to higher levels of resource intensity). Each organization in the coalition is asked to report on the extent to which they interact with the other coalition members along four dimensions: (1) process activities in which partners engage in simple ways such as attending meetings together; (2) cooperative activities in which partners engage in process activities as well as share information about their own activities; (3) coordinated activities which include cooperative activities as well as those in which data are shared, trainings are coordinated, and interventions are developed; and (4) integrated activities where the partners engage in coordinated activities and jointly implement activities such as trainings.
- Hours spent on coalition activities by partner organization staff.
- We ascertained the number of participants in each coalition and assigned each to one of the 11 CDC sectors. To identify the number of sectors we simply counted the number of sectors represented in each coalition (subtracting duplicates).

To assess how coalition members interact to achieve their community resilience goals we adapted the PARTNER survey to include questions about the types of activities engaged in by the coalitions. Drawing on U.S. CDC Public Health Emergency Preparedness (PHEP) or community preparedness metric guidance [11] and community resilience goals [4], we measured activities that can be considered intermediate capacities relative to resilience such as developing plans to communicate with residents during a disaster and developing integrated emergency plans for coalition partners. We also measured activities related to resilience outcomes such as exercising or implementing communication and disaster plans [34].

Disasters did not occur during the study period; thus, it is difficult to truly assess resilience-related outcomes other than through exercises (see Chandra and colleagues [35] for a discussion of our resilience tabletop). Therefore, to assess progress toward resilience, we chose to measure intermediate activities at the community-coalition level, such as leveraging resources, developing programs, and strengthening community partnerships. These are central to the four resilience levers described earlier. Together, these activities describe the capacity of the coalition to work together to achieve its goals and support the larger public health infrastructure. The Intermediate Resilience Activities are measured by four items asking whether the coalition has:

- Worked with media to communicate about coalition activities;
- Developed a plan to communicate with residents during a disaster (e.g., door-to-door, phone tree, rapid language translation);
- Exercised or implemented a community disaster plan during an emergency;
- Exercised or implemented a disaster communication plan during a disaster.

In addition, we assessed whether the coalitions engaged in activities considered to be Standard Disaster Preparedness:

- Made or translated disaster materials (e.g., brochures, posters, etc.);
- Put disaster brochures or other materials into the community;
- Organized community events such as health fairs, or convened neighborhood watch groups.

Finally, we assessed whether the coalition implemented activities related to the Community Resilience Toolkit Training, including whether the community engaged in:

- Community leadership training;
- Psychological first aid training;
- Community Emergency Response Team (CERT) training;
- Community health worker training;
- Community mapping events to assess vulnerabilities and hazards;
- Development of integrated emergency plans for coalition partners with roles and responsibilities defined.

### 2.3. Data Analysis

The unit of analysis was the network within the community coalitions which included the organizations and people (e.g., local faith-based groups, emergency managers, and businesses) involved as members of the coalitions. We also considered as a dependent variable the amount of time individual coalition participants spent on work related to a collaborative.

To examine how partnerships were formed between LACDPH and community partners, we present descriptive analysis of the networks formed using Social Network Analysis (SNA) including network characteristics such as density, trust, and value which indicate how well connected the organizations that comprise each coalition are with one another, as well as individual ratings about how important each organization is to the coalition. To assess the characteristics of partnerships, and how these changed over the course of the study period, we summarized both the number of organizations involved in each coalition, and the sector associated with each organization. We then compared these across study arms and year. We measured activities by respondents’ reported activities (e.g., developing disaster communication plans, conducting psychological first aid trainings), and by the extent to which they reported the coordination of these activities among partners along the continuum of lower coordination (e.g., process activities) to greater coordination (e.g., integrated activities). In this way, we assessed not just whether the organizations were involved in the partnership, but also the depth of their involvement.

Where possible, we use a t-statistic to compare mean differences between community resilience and preparedness coalitions. The t-statistic is used to compare means of groups with small sample sizes. A large, positively-valued t-statistic indicates that the resilience coalitions reported higher values for that characteristic. Significance was set at *p* < 0.05 for all analyses.

## 3. Results

### 3.1. Interactions among Coalition Members

In year 1, resilience communities had more partners (10 on average) in their coalition than preparedness communities (7 on average), but the difference was not statistically significant (*p* = 0.12). In year 2, coalitions in both the resilience and preparedness communities continued to add more organizations. The coalitions in resilience communities remained larger, averaging 15 organizations, compared to an average of 8 organizations in preparedness coalitions. The year 2 difference approached significance (*p* = 0.06). While the number of organizations involved in the coalitions grew, the average number of sectors that these organizations represented also increased over the two years, with resilience communities reporting a significantly higher number of sectors involved in their coalitions in both years (*p* = 0.03 and *p* > 0.001 respectively) (Table 1).

Resilience communities started the study with lower levels of trust among coalition members compared to the preparedness communities at baseline (*p* > 0.01). By year 2, trust among the coalition members in the resilience communities had increased more than it had in the preparedness communities so that it was equivalent at follow-up. There were no differences in perceived value of coalition member contributions to the network between resilience and preparedness communities in either year (Table 1).

The resilience communities reported higher density (more connections) than the preparedness communities in the first year (*p* = 0.04). However, the difference in density was not statistically significant in the second year (Table 1). There were no statistically significant differences in the average number of hours spent working on coalition activities between community types in either year (Table 1).

### 3.2. Activities Conducted by Coalitions

At follow-up, we asked the respondents to report on the types of activities they conducted as part of their coalition. Of the 14 key activities assessed, the responding organizations in the resilience communities were more likely than those in preparedness communities to report only one Intermediate Resilience Activity: communicating about coalition activities through the media. Resilience coalitions were also more likely to report conducting five Community Resilience Toolkit Training activities; community mapping of assets and vulnerabilities, identifying priority hazards, providing psychological first aid training, developing integrated emergency plans with partners and holding CERT trainings. Respondents in preparedness communities reported greater involvement than resilience communities in six activities (Table 2). These included one Standard Disaster Preparedness Activity: making or translating disaster materials; three Intermediate Resilience Activities: a plan to communicate with residents during a disaster, exercising a community disaster plan, exercising a disaster communication plan; and two Community Resilience Toolkit Training activities: holding community leadership trainings, holding community health worker trainings.

### 3.3. Coordination

Relationships among collaboration members are measured on a continuum of activity coordination ranging from lower to higher resource intensive activities (e.g., process to integrated). On average (and in both years), coalition members in both types of communities tended to have greater process and cooperative relationships than coordinated or integrated relationships (Table 3). Process activities decreased in year 2 in both community types, and Integrated activities increased (although Integrated activities increased by a greater percent among preparedness communities). Within resilience communities, cooperative activities are most often reported in year 2; Integrated activities were next frequent. Within the preparedness communities, Integrated activities were most often reported in year 2, with the next frequently reported activities being cooperative. Across community types and years, coordinated activities were consistently the lowest reported activity (Table 3).

## 4. Discussion

This research provides, to our knowledge, the first use of partnership metrics to measure changes in resilience capacities associated with different approaches to supporting organizational partnerships and networks. We focused on the quality of relationships, the activities that were conducted, and the level of coordination of partners in the activities. While we identified few substantial differences between the resilience and preparedness arms, there were several lessons learned that can inform LHDs and other agencies interested in engaging communities and community-based organizations to improve community resilience.

Resilience and preparedness communities reported engaging in different types of activities. Five of the six activities that resilience communities conducted at higher percentages than preparedness communities were activities on which they had received training during the project. Thus, we would expect them to implement more of these activities. Further, the resilience coalitions were larger and had more varied partners, which may be due at least in part to the training and encouragement they received to expand their coalitions. It is possible that these broader coalitions contributed to stronger resilience capabilities, since integrating across sectors in a community is a hallmark of a more robust and resilient community network. As reported in a previous paper, the resilience coalitions reported conducting 58 trainings compared to 18 conducted by the preparedness coalitions [36]. Twenty of the resilience coalitions’ 58 trainings were to vulnerable population groups (defined as groups in the community possessing a culture, language, or other distinguishing characteristic that places them at higher risk in a disaster) compared to four of the preparedness coalitions’ 18 trainings. Also, many of these 58 trainings were of the train-the-trainer model. Thus, the resilience coalitions may have made considerable impact with these “high-touch” activities [36]. In contrast, many of the activities that preparedness coalition members reported conducting were standard practice for community-based emergency preparedness such as creating or translating brochures, or were related to standard practice such as exercising or implementing community disaster plans during an emergency, and exercising or implementing a communication plan during a disaster. This corresponds to earlier reported findings in which the preparedness communities engaged in more “low-touch” activities such as handing out brochures at health fairs [36]. It is notable that a few of the preparedness coalitions found their way to conducting some resilience activities on their own, demonstrating their intuitive appeal.

The findings of this study offer examples and benchmarks for types of partnership development and activities that may promote a community resilience perspective in coalitions focusing on disaster preparedness. Key features of LACCDR interventions that may have promoted a resilience approach include having a specific toolkit of resilience activities, accompanied by training and facilitation provided by disaster-trained, public health nurses. However, it is clear that some coalitions that do not receive such training may also adopt some of these resilience-focused activities. The finding that preparedness communities were more likely to report conducting community leadership and health worker training shows some evidence for this, and suggests that the simple process of connecting organizations to the health department and engaging them in discussions about disaster preparedness may be sufficient for some communities to develop collaborative activities that support resilience.

While resilience communities had lower trust at baseline, which may have been an artifact of the random assignment or alternatively of initially having larger coalitions, trust within the resilience coalitions improved within one year. It is very likely that as these organizations worked together over time and their staff became familiar with one another, they increased their trust in their partners’ capacity to be reliable participants in the coalition’s activities. The process of building resilience coalitions, which calls for a greater number and diversity of partners, may be therefore initially disruptive. But, over time trust may improve within such larger coalitions. We also saw increases in the amount of integrated activities that coalition members were doing together, suggesting that creating trusting relationships can be a platform for productive activity. All of this suggests that a community resilience approach may benefit from a longer time course and more attention to relationship-building to develop trust and goals than the traditional preparedness approach.

There are several limitations to this study. The first is that the differences in size of the coalitions could have contributed to differences in the types of activities the coalitions sponsored. Resilience communities started with larger coalitions and therefore may have had greater capacity to engage in activities on which they were trained. The specific resources contributed by coalitions’ members across the two arms of the study were not systematically measured. This makes it difficult to tell whether one type of coalition was better prepared to engage in activities that required greater resources or more specialized partners. The fact that both coalition types grew over the course of the study suggests that the coalitions recognized a need to add partners to accomplish their goals, or that they found the coalition structure engaging, thus encouraging them to add new members. Further, one year may not be long enough to observe differences in approaches, relationships, and partnered activities between intervention arms. The resilience communities engaged in training and other activities that may have slowed their initial progress but increased their capacity for future activities. This idea is also supported by prior research on these communities which suggests that the community resilience coalitions recognized the importance of relationship building, making it a priority [36,37]. We do not have data on whether or how activities in both coalition types continued to evolve. LACCDR was a demonstration project and while it was being fielded, the investigators were involved in creating the approach and toolkits as well as supporting implementation and evaluation. This aspect of LACCDR resulted in delays which may have created inconsistencies in the framing of resilience for resilience coalitions which, in turn, may have blunted comparisons attributable to study arm (community resilience versus preparedness).

Another limitation of this study is the lack of pre-identified and validated community resilience outcome measures. Performance measurement in public health is an important issue because it is a critical part of evaluating whether public health (or a public health department more specifically) is achieving its anticipated health outcomes [38]. One of the key challenges of measuring community resilience resides in the relative rarity of emergency events, making it difficult to establish an evidence base for validating a set of measures. However, tabletop exercises developed as part of this LACCDR work, may provide new opportunity to stress test some of these partnership capabilities [30]. In addition, our surveys did not measure differences in the quality of activities. Thus, simple counts may not measure actual differences in resilience. As Bromley and colleagues [33] reported, the resilience and preparedness coalitions took very different approaches to community education, with preparedness communities opting for more passive activities (passing out brochures) compared to resilience coalitions’ more active style of attempting to engage and learn from community members to shape their activities and approach. This study is also limited by its short duration. This, combined with our measurement focus to assess the adoption of specific activities in each coalition, means that that we cannot evaluate the extent to which there may have been differences in how coalitions adopt a community development approach in which communities are empowered to maintain ownership over the activities and approaches they apply to building community resilience over time [39]. In addition, the study ended prior to some of the more recent flooding and wildfire events which have affected Los Angeles County. Thus, we cannot ascertain how the different types of communities reacted to these events. Finally, the study may be limited by both small sample sizes and misclassification of activities, where some respondents may have positively indicated that they accomplished the same thing (e.g., community health worker training) but could have been referring to completely different activities, further, recall bias may have affected their own accounts of the activities in which they engaged.

## 5. Conclusions

In short, we found that it is possible to measure one of the key characteristics of resilience, namely the growth of organizational networks and partnerships over time. In addition, the differences in the types of activities in which resilience communities reported engaging are directly related to the trainings they undertook within the project, suggesting that initial engagement in training does relate to use and application of specific preparedness approaches, whether standard or resilience-building, even with some overlap across arms. Information gleaned from this research may be useful to LHDs interested in applying a community resilience framework to their disaster preparedness work, as well as offer a framework that may have applicability to the use of coalitions more broadly in public health practice, a key issue for future research and public health practice demonstrations.

## Figures and Tables

**Table 1:** Differences in Partnership Characteristics by Community Type and Year.

Partnership Characteristics	Year 1			Year 2		
	Resilience	Preparedness	*p*-Value	Resilience	Preparedness	*p*-Value
Mean number of sectors per coalition	6	4	0.03	7	4	>0.001
Mean number of organizations per coalition	10	7	0.12	15	8	0.06
Mean reported trust	2.91	3.43	>0.01	3.24	3.37	0.50
Mean reported value	2.97	3.20	0.37	2.88	3.05	0.52
Mean relative density	0.72	0.54	0.04	0.60	0.75	0.14
Mean hours spent per month on coalition activities	12.56	13.4	0.92	27.79	31.56	0.73

**Table 2:** Reported Activities by Coalition Type and Year.

Activities Completed	Community Type	
	Preparedness	Resilience
Made or translated disaster materials (e.g., brochures, posters, etc.)	86%	75%
Put disaster brochures or other materials into the community	100%	100%
Worked with the media (radio, tv, newspapers) to communicate about coalition activities	43%	50%
Developed plan to communicate with residents during a disaster	86%	63%
Developed integrated emergency plans for coalition partners	43%	63%
Participated in community mapping	29%	100%
Identified priority hazards in the community	86%	100%
Organized community events (e.g., health fairs, convening neighborhood watch)	100%	100%
Exercised or implemented community disaster plan during an emergency	57%	25%
Exercised or implemented disaster communication plan during a disaster	43%	25%
Held community leadership training	71%	63%
Held psychological first aid training	14%	38%
Held Community Emergency Response Team (CERT) training	86%	88%
Held community health worker training	29%	0%

**Table 3:** Activity Types by Community Type and Year.

Mean Activity Type among Coalition Partners	Preparedness (%)		Resilience (%)	
	Year 1	Year 2	Year 1	Year 2
Process	34.34	18.66	23.74	19.15
Cooperative	29.27	36.72	39.18	44.87
Coordinated	9.68	5.28	15.68	11.90
Integrated	26.71	39.33	21.27	24.09
